# Supplementary material for: A Likelihood Approach for Real-Time Calibration of Stochastic Compartmental Epidemic Models
Source: PLoS Comput Biol. 2017 Jan 17;13(1):e1005257. doi: 10.1371/journal.pcbi.1005257 (PMC5240920; doi:10.1371/journal.pcbi.1005257)
Supplement: S1 File — (TAR.GZ) [file pcbi.1005257.s014.tar.gz › HSPH_Online-SI-Revision/IPoi09/n1-extreme/n1-extreme_BENCH2_table8.pdf]

1	newly 3 weeks spec	5.	7.67164	{2., 18.}	94.0299	0.288398	newly 3 weeks cumul	23.	31.9701	{8., 76.}	78.196	0.262068	newly 5 weeks spec	0.1	3.61194	{0., 7.}	3538.81	1.27821	newly 5 weeks cumul	25.	40.5821	{11., 88.}	92.5373	0.263791	newly 52 weeks cumul	29.	48.4478	{12., 101.}	96.5003	0.267095
2	newly 3 weeks spec	4.	10.8361	{4., 15.}	178.279	0.350253	newly 3 weeks cumul	26.	44.1311	{18., 65.}	83.4805	0.218624	newly 5 weeks spec	4.	6.45902	{2., 11.}	93.4426	0.276551	newly 5 weeks cumul	34.	58.7705	{24., 88.}	84.812	0.215437	newly 48 weeks cumul	44.	80.9344	{35., 115.}	94.1505	0.227242
3	newly 3 weeks spec	13.	18.0164	{7., 29.}	64.3127	0.210133	newly 3 weeks cumul	58.	69.918	{35., 111.}	47.7388	0.16243	newly 5 weeks spec	7.	10.8361	{4., 15.}	83.3724	0.240362	newly 5 weeks cumul	73.	96.0328	{46., 155.}	54.278	0.169349	newly 50 weeks cumul	89.	132.836	{60., 201.}	65.9422	0.183089
4	newly 3 weeks spec	10.	18.0159	{7., 33.}	98.5714	0.260485	newly 3 weeks cumul	62.	68.7778	{30., 116.}	48.9247	0.198101	newly 5 weeks spec	6.	10.2063	{3., 18.}	92.8571	0.259934	newly 5 weeks cumul	83.	92.4921	{39., 150.}	50.5259	0.202505	newly 49 weeks cumul	123.	130.206	{52., 211.}	53.2714	0.226007
5	newly 3 weeks spec	9.	0.	{0., 0.}	100.	1.95424	newly 3 weeks cumul	33.	0.	{0., 0.}	100.	2.51851	newly 5 weeks spec	4.	0.	{0., 0.}	100.	1.60206	newly 5 weeks cumul	44.	0.	{0., 0.}	100.	2.64345	newly 42 weeks cumul	59.	0.	{0., 0.}	100.	2.77085
6	newly 3 weeks spec	11.	13.5672	{4., 29.}	72.7273	0.279921	newly 3 weeks cumul	43.	50.3582	{18., 94.}	61.5411	0.229963	newly 5 weeks spec	6.	8.08955	{2., 16.}	76.6169	0.30023	newly 5 weeks cumul	53.	69.1045	{26., 123.}	66.7699	0.221256	newly 47 weeks cumul	71.	96.0597	{34., 178.}	72.6298	0.232046
7	newly 3 weeks spec	6.	8.94521	{3., 15.}	75.1142	0.254109	newly 3 weeks cumul	35.	36.9863	{15., 69.}	47.4755	0.198789	newly 5 weeks spec	5.	4.90411	{1., 9.}	54.5205	0.315935	newly 5 weeks cumul	52.	48.3973	{20., 92.}	45.0738	0.215876	newly 51 weeks cumul	64.	62.3699	{25., 113.}	46.0402	0.211617
8	newly 3 weeks spec	9.	7.81356	{1., 15.}	65.5367	0.422962	newly 3 weeks cumul	27.	29.5254	{7., 56.}	71.3748	0.312065	newly 5 weeks spec	1.	5.01695	{0., 11.}	432.203	0.648014	newly 5 weeks cumul	32.	40.8136	{10., 77.}	81.6737	0.302803	newly 45 weeks cumul	35.	58.9831	{14., 107.}	107.264	0.301698
9	newly 3 weeks spec	3.	5.01587	{1., 11.}	104.233	0.368641	newly 3 weeks cumul	16.	22.7143	{7., 48.}	76.8849	0.251414	newly 5 weeks spec	3.	2.5873	{0., 5.}	67.7249	0.478141	newly 5 weeks cumul	22.	29.0159	{9., 55.}	70.4185	0.244505	newly 52 weeks cumul	25.	34.619	{11., 69.}	71.873	0.233307
10	newly 3 weeks spec	17.	10.8246	{2., 22.}	65.8411	0.48851	newly 3 weeks cumul	46.	37.1579	{10., 75.}	64.836	0.36916	newly 5 weeks spec	5.	6.24561	{0., 13.}	95.7895	0.476837	newly 5 weeks cumul	57.	51.3158	{13., 99.}	66.8513	0.3431	newly 45 weeks cumul	77.	74.9298	{19., 134.}	71.6792	0.347784
11	newly 3 weeks spec	5.	6.0411	{1., 12.}	69.589	0.311375	newly 3 weeks cumul	17.	24.5342	{9., 54.}	68.1708	0.225292	newly 5 weeks spec	0.1	2.80822	{0., 6.}	2732.88	1.22648	newly 5 weeks cumul	19.	31.5342	{11., 69.}	83.5616	0.245351	newly 51 weeks cumul	19.	38.1233	{13., 75.}	112.329	0.292497
12	newly 3 weeks spec	1.	9.90278	{2., 19.}	893.056	0.880912	newly 3 weeks cumul	17.	43.9028	{18., 79.}	160.866	0.348522	newly 5 weeks spec	2.	5.30556	{1., 12.}	181.944	0.400775	newly 5 weeks cumul	22.	56.2222	{23., 111.}	158.586	0.343427	newly 52 weeks cumul	22.	69.2917	{27., 140.}	216.225	0.420483
13	newly 3 weeks spec	20.	12.	{3., 24.}	59.8462	0.419434	newly 3 weeks cumul	80.	45.1077	{16., 88.}	57.5385	0.407527	newly 5 weeks spec	17.	7.49231	{1., 17.}	68.7783	0.610194	newly 5 weeks cumul	116.	61.7077	{20., 115.}	59.9072	0.431819	newly 45 weeks cumul	196.	94.4308	{28., 181.}	65.3846	0.492184
14	newly 3 weeks spec	2.	5.52703	{1., 12.}	192.568	0.436015	newly 3 weeks cumul	9.	23.9459	{9., 42.}	173.574	0.379727	newly 5 weeks spec	0.1	3.21622	{0., 7.}	3148.65	1.23774	newly 5 weeks cumul	9.	31.4595	{12., 53.}	252.252	0.471668	newly 49 weeks cumul	9.	40.4865	{14., 73.}	351.351	0.568851
15	newly 3 weeks spec	2.	12.2857	{4., 23.}	514.286	0.6743	newly 3 weeks cumul	21.	46.6429	{17., 104.}	131.973	0.297797	newly 5 weeks spec	1.	7.76786	{1., 15.}	680.357	0.732965	newly 5 weeks cumul	25.	63.8393	{22., 138.}	162.929	0.340335	newly 44 weeks cumul	29.	96.9643	{27., 215.}	240.394	0.429037
16	newly 3 weeks spec	13.	5.75714	{1., 11.}	60.3297	0.524957	newly 3 weeks cumul	45.	24.8571	{9., 43.}	51.619	0.35893	newly 5 weeks spec	6.	3.31429	{0., 6.}	57.1429	0.51535	newly 5 weeks cumul	59.	32.4857	{12., 53.}	52.155	0.36405	newly 51 weeks cumul	82.	41.1429	{15., 64.}	55.4704	0.399748
17	newly 3 weeks spec	14.	14.871	{4., 27.}	58.7558	0.252948	newly 3 weeks cumul	63.	59.6935	{27., 109.}	50.	0.227893	newly 5 weeks spec	8.	10.0645	{2., 20.}	74.1935	0.275604	newly 5 weeks cumul	82.	81.5645	{36., 142.}	52.262	0.226081	newly 48 weeks cumul	132.	114.565	{47., 196.}	54.1178	0.267901
18	newly 3 weeks spec	15.	8.01316	{3., 12.}	57.9825	0.409584	newly 3 weeks cumul	37.	31.7763	{12., 54.}	43.4211	0.225182	newly 5 weeks spec	5.	4.17105	{1., 8.}	53.9474	0.378374	newly 5 weeks cumul	50.	41.4605	{16., 71.}	43.7632	0.235436	newly 51 weeks cumul	60.	53.4211	{20., 99.}	43.4211	0.222682
19	newly 3 weeks spec	10.	8.63514	{3., 15.}	48.7838	0.254756	newly 3 weeks cumul	28.	35.8784	{15., 58.}	54.1988	0.183189	newly 5 weeks spec	4.	4.78378	{1., 8.}	62.1622	0.302114	newly 5 weeks cumul	43.	47.1351	{20., 86.}	47.3916	0.189705	newly 52 weeks cumul	65.	60.4189	{25., 102.}	44.9688	0.212534
20	newly 3 weeks spec	4.	8.5	{2., 20.}	139.113	0.349848	newly 3 weeks cumul	15.	32.7097	{11., 73.}	131.828	0.306885	newly 5 weeks spec	2.	4.27419	{1., 8.}	137.903	0.354676	newly 5 weeks cumul	21.	43.2903	{14., 87.}	119.508	0.283086	newly 45 weeks cumul	30.	59.8871	{19., 113.}	114.14	0.270548
21	newly 3 weeks spec	20.	21.8448	{7., 42.}	61.1207	0.265619	newly 3 weeks cumul	78.	79.6724	{30., 150.}	50.1989	0.226678	newly 5 weeks spec	5.	14.6379	{3., 31.}	204.483	0.387189	newly 5 weeks cumul	98.	111.379	{40., 209.}	56.2984	0.226972	newly 48 weeks cumul	119.	170.19	{51., 312.}	76.8038	0.242616
22	newly 3 weeks spec	4.	3.8	{1., 8.}	61.	0.36144	newly 3 weeks cumul	28.	17.1733	{6., 34.}	51.4286	0.345212	newly 5 weeks spec	5.	2.30667	{0., 5.}	64.	0.680274	newly 5 weeks cumul	41.	22.8533	{8., 40.}	53.4959	0.375777	newly 49 weeks cumul	59.	29.3333	{10., 50.}	57.2429	0.416639
23	newly 3 weeks spec	8.	8.63889	{3., 15.}	56.9444	0.246109	newly 3 weeks cumul	27.	37.0972	{14., 68.}	59.5165	0.192869	newly 5 weeks spec	5.	5.31944	{1., 9.}	56.3889	0.303529	newly 5 weeks cumul	38.	49.3333	{18., 92.}	55.117	0.190126	newly 51 weeks cumul	59.	63.5833	{23., 113.}	47.4105	0.197233
24	newly 3 weeks spec	5.	5.57377	{2., 11.}	56.7213	0.234687	newly 3 weeks cumul	19.	25.4098	{10., 41.}	64.2796	0.221429	newly 5 weeks spec	2.	3.09836	{1., 6.}	90.9836	0.340956	newly 5 weeks cumul	25.	32.4262	{14., 50.}	61.3115	0.214491	newly 52 weeks cumul	25.	38.2459	{15., 69.}	76.3279	0.227528
25	newly 3 weeks spec	8.	15.5968	{5., 36.}	110.282	0.280036	newly 3 weeks cumul	31.	61.	{27., 111.}	103.746	0.259215	newly 5 weeks spec	8.	10.3226	{3., 20.}	68.9516	0.253249	newly 5 weeks cumul	48.	82.871	{36., 157.}	85.4167	0.229452	newly 49 weeks cumul	114.	115.823	{47., 224.}	51.8817	0.231577
26	newly 3 weeks spec	4.	6.94286	{3., 15.}	95.	0.275836	newly 3 weeks cumul	13.	32.6	{14., 62.}	155.604	0.344366	newly 5 weeks spec	1.	3.84286	{1., 7.}	298.571	0.536138	newly 5 weeks cumul	14.	41.6143	{18., 76.}	200.51	0.406777	newly 51 weeks cumul	19.	50.8143	{19., 111.}	171.654	0.364104
27	newly 3 weeks spec	15.	11.069	{2., 24.}	68.5057	0.44002	newly 3 weeks cumul	54.	42.7759	{12., 85.}	60.7599	0.342161	newly 5 weeks spec	8.	7.65517	{1., 15.}	69.8276	0.41455	newly 5 weeks cumul	70.	61.1552	{17., 116.}	62.9803	0.326847	newly 46 weeks cumul	110.	90.5172	{24., 168.}	65.5799	0.362025
28	newly 3 weeks spec	9.	10.2105	{3., 19.}	58.4795	0.260908	newly 3 weeks cumul	43.	42.2237	{16., 85.}	48.6842	0.223977	newly 5 weeks spec	3.	5.38158	{1., 12.}	110.965	0.377082	newly 5 weeks cumul	53.	55.3158	{20., 110.}	49.0566	0.212227	newly 50 weeks cumul	70.	71.2105	{24., 142.}	49.0602	0.221248
29	newly 3 weeks spec	2.	11.831	{3., 22.}	502.817	0.69764	newly 3 weeks cumul	14.	47.7183	{16., 96.}	241.65	0.42872	newly 5 weeks spec	0.1	8.12676	{3., 17.}	8029.58	1.76787	newly 5 weeks cumul	14.	66.1831	{24., 129.}	372.736	0.568519	newly 47 weeks cumul	17.	92.0563	{31., 199.}	441.508	0.618423
30	newly 3 weeks spec	2.	4.48571	{0., 10.}	160.	0.4356	newly 3 weeks cumul	16.	15.9571	{3., 32.}	66.1607	0.339117	newly 5 weeks spec	3.	3.11429	{0., 7.}	87.619	0.555	newly 5 weeks cumul	21.	22.6286	{6., 48.}	67.3469	0.321854	newly 42 weeks cumul	31.	32.5857	{7., 72.}	71.659	0.335909
31	newly 3 weeks spec	4.	5.77941	{1., 13.}	79.0441	0.266497	newly 3 weeks cumul	13.	24.3971	{9., 44.}	103.959	0.272335	newly 5 weeks spec	3.	2.76471	{0., 6.}	66.6667	0.439151	newly 5 weeks cumul	20.	31.1618	{11., 57.}	78.4559	0.238101	newly 51 weeks cumul	27.	38.9265	{14., 69.}	68.7908	0.22122
32	newly 3 weeks spec	1.	8.32813	{2., 16.}	739.063	0.823023	newly 3 weeks cumul	8.	37.2031	{18., 60.}	365.82	0.582523	newly 5 weeks spec	0.1	4.29688	{1., 9.}	4209.38	1.43931	newly 5 weeks cumul	8.	47.8906	{21., 74.}	498.633	0.687588	newly 51 weeks cumul	10.	58.5313	{25., 97.}	485.313	0.681561
33	newly 3 weeks spec	21.	14.7966	{3., 31.}	59.887	0.375348	newly 3 weeks cumul	67.	53.661	{18., 100.}	55.5274	0.311059	newly 5 weeks spec	11.	10.5763	{2., 22.}	71.3405	0.380858	newly 5 weeks cumul	89.	75.3729	{25., 143.}	59.0745	0.312605	newly 47 weeks cumul	124.	112.644	{32., 209.}	63.8054	0.319184
34	newly 3 weeks spec	9.	10.1299	{3., 20.}	60.7504	0.26369	newly 3 weeks cumul	31.	42.0519	{16., 83.}	61.8768	0.203981	newly 5 weeks spec	2.	5.45455	{1., 12.}	185.714	0.396703	newly 5 weeks cumul	36.	55.5584	{22., 106.}	72.0779	0.209286	newly 50 weeks cumul	40.	71.5584	{29., 139.}	91.6883	0.240524
35	newly 3 weeks spec	11.	9.86667	{3., 19.}	54.1818	0.278916	newly 3 weeks cumul	46.	40.36	{15., 78.}	47.971	0.240284	newly 5 weeks spec	5.	5.29333	{1., 9.}	57.6	0.320491	newly 5 weeks cumul	59.	53.3867	{20., 102.}	48.0226	0.234768	newly 51 weeks cumul	68.	68.4667	{24., 128.}	49.7843	0.22159
36	newly 3 weeks spec	10.	14.6825	{5., 23.}	75.0794	0.224462	newly 3 weeks cumul	26.	56.8571	{25., 106.}	122.222	0.278563	newly 5 weeks spec	7.	9.4127	{3., 19.}	74.3764	0.269317	newly 5 weeks cumul	37.	77.619	{32., 130.}	114.843	0.262352	newly 48 weeks cumul	53.	108.746	{45., 183.}	112.489	0.251677
37	newly 3 weeks spec	11.	10.5818	{3., 23.}	53.3884	0.2667	newly 3 weeks cumul	35.	40.6182	{15., 74.}	61.039	0.24028	newly 5 weeks spec	7.	7.63636	{1., 16.}	77.6623	0.430475	newly 5 weeks cumul	50.	56.4727	{19., 109.}	61.9636	0.251428	newly 43 weeks cumul	55.	90.6727	{24., 172.}	91.5041	0.255662
38	newly 3 weeks spec	6.	8.18033	{3., 12.}	70.2186	0.228456	newly 3 weeks cumul	20.	34.2951	{14., 49.}	85.4098	0.216296	newly 5 weeks spec	2.	4.96721	{1., 8.}	171.311	0.405558	newly 5 weeks cumul	28.	45.7213	{19., 70.}	79.3326	0.205368	newly 48 weeks cumul	40.	62.918	{26., 89.}	77.9508	0.2098
39	newly 3 weeks spec	6.	8.56579	{3., 15.}	70.8333	0.239963	newly 3 weeks cumul	22.	33.3158	{13., 56.}	71.4115	0.218492	newly 5 weeks spec	2.	4.28947	{1., 7.}	136.842	0.374314	newly 5 weeks cumul	28.	43.3553	{15., 79.}	74.5771	0.227068	newly 51 weeks cumul	34.	55.8421	{20., 100.}	83.7461	0.247011
40	newly 3 weeks spec	20.	5.78788	{1., 14.}	72.4242	0.736945	newly 3 weeks cumul	45.	25.8182	{8., 49.}	58.9899	0.399814	newly 5 weeks spec	5.	3.39394	{0., 7.}	63.6364	0.563293	newly 5 weeks cumul	56.	33.2424	{11., 64.}	58.3874	0.389016	newly 49 weeks cumul	62.	42.3939	{14., 82.}	57.3314	0.353145
41	newly 3 weeks spec	17.	12.2857	{4., 23.}	56.9328	0.33842	newly 3 weeks cumul	35.	46.6429	{17., 104.}	67.2449	0.225525	newly 5 weeks spec	9.	7.76786	{1., 15.}	66.0714	0.383629	newly 5 weeks cumul	50.	63.8393	{22., 138.}	64.5357	0.22441	newly 44 weeks cumul	61.	96.9643	{27., 215.}	86.3583	0.246741
42	newly 3 weeks spec	1.	4.84722	{1., 12.}	401.389	0.614778	newly 3 weeks cumul	20.	22.9722	{7., 55.}	68.75	0.288303	newly 5 weeks spec	1.	2.91667	{0., 7.}	225.	0.514336	newly 5 weeks cumul	23.	29.3056	{8., 65.}	73.6715	0.273589	newly 52 weeks cumul	25.	34.6944	{9., 77.}	80.7778	0.275534
43	newly 3 weeks spec	13.	13.8158	{5., 27.}	53.6437	0.23616	newly 3 weeks cumul	49.	57.7763	{28., 114.}	49.1139	0.18632	newly 5 weeks spec	1.	8.52632	{2., 15.}	755.263	0.828051	newly 5 weeks cumul	57.	77.0789	{33., 142.}	57.2022	0.189612	newly 51 weeks cumul	68.	99.4079	{39., 184.}	66.041	0.207238
44	newly 3 weeks spec	6.	14.8548	{6., 25.}	153.495	0.325013	newly 3 weeks cumul	30.	54.7419	{23., 86.}	92.6882	0.233121	newly 5 weeks spec	3.	7.82258	{3., 11.}	168.28	0.339001	newly 5 weeks cumul	42.	73.2742	{30., 102.}	85.4455	0.216087	newly 49 weeks cumul	55.	101.	{43., 144.}	94.1935	0.229546
45	newly 3 weeks spec	9.	16.4545	{6., 29.}	98.1241	0.268159	newly 3 weeks cumul	41.	67.5455	{29., 135.}	78.9357	0.226391	newly 5 weeks spec	11.	9.79221	{3., 18.}	49.7048	0.26206	newly 5 weeks cumul	64.	89.4805	{34., 172.}	62.1753	0.202568	newly 52 weeks cumul	78.	113.922	{46., 214.}	67.699	0.215289
46	newly 3 weeks spec	12.	8.18033	{3., 12.}	56.9672	0.345925	newly 3 weeks cumul	35.	34.2951	{14., 49.}	51.3817	0.221049	newly 5 weeks spec	5.	4.96721	{1., 8.}	63.6066	0.349575	newly 5 weeks cumul	52.	45.7213	{19., 70.}	51.3556	0.243548	newly 49 weeks cumul	69.	62.918	{26., 89.}	52.1026	0.241373
47	newly 3 weeks spec	16.	18.0164	{7., 29.}	50.3074	0.200014	newly 3 weeks cumul	65.	69.918	{35., 111.}	44.1866	0.169595	newly 5 weeks spec	11.	10.8361	{4., 15.}	57.8241	0.262814	newly 5 weeks cumul	87.	96.0328	{46., 155.}	47.0134	0.175874	newly 50 weeks cumul	118.	132.836	{60., 201.}	49.8055	0.18221
48	newly 3 weeks spec	17.	15.1148	{6., 25.}	51.5911	0.257661	newly 3 weeks cumul	49.	55.5738	{23., 86.}	50.4851	0.189121	newly 5 weeks spec	7.	8.	{3., 12.}	61.1241	0.241765	newly 5 weeks cumul	64.	74.3443	{31., 102.}	50.999	0.181997	newly 50 weeks cumul	85.	102.77	{44., 144.}	54.6191	0.187324
49	newly 3 weeks spec	8.	3.91803	{1., 8.}	62.5	0.580897	newly 3 weeks cumul	31.	17.0328	{6., 27.}	55.3146	0.383657	newly 5 weeks spec	3.	2.01639	{0., 6.}	68.8525	0.642842	newly 5 weeks cumul	39.	22.1475	{9., 34.}	53.9723	0.363388	newly 52 weeks cumul	53.	25.9508	{9., 45.}	58.3978	0.423346
50	newly 3 weeks spec	6.	3.49231	{0., 7.}	61.7949	0.560639	newly 3 weeks cumul	38.	13.8462	{4., 26.}	67.2874	0.564849	newly 5 weeks spec	6.	1.92308	{0., 5.}	70.5128	0.881435	newly 5 weeks cumul	47.	18.4615	{6., 33.}	65.9574	0.542518	newly 50 weeks cumul	63.	23.4462	{8., 44.}	67.619	0.567729
